# From Experience to Evangelism: Emotional and Social Drivers of Online Cosmetics Purchase Behavior—A 4Es Perspective

**DOI:** 10.3390/bs16071054

**Published:** 2026-06-24

**Authors:** Kris Jangjarat, Pongsakorn Limna, Yarnaphat Shaengchart

**Affiliations:** 1 International College, Pathumthani University, Mueang 12000, Pathum Thani, Thailand; kris.j@ptu.ac.th; 2 Faculty of Information Technology and Digital Innovation, King Mongkut’s University of Technology North Bangkok, Bangsue, Bangkok 10800, Thailand

**Keywords:** 4Es framework (Experience, Exchange, Everyplace, and Evangelism), online cosmetics purchase behavior, digital consumer engagement, Thailand

## Abstract

This study examines how the 4Es framework—Experience, Exchange, Everyplace, and Evangelism—influences online cosmetics purchase behavior in Thailand, addressing the growing importance of digital consumer engagement in emerging markets. A mixed-methods approach was employed, combining quantitative data from a structured survey with qualitative insights from semi-structured interviews. Binary logistic regression analysis was used to assess the effects and predictive power of the 4Es and selected demographic and behavioral variables. The results indicate that all four dimensions significantly influence purchase behavior, with Evangelism emerging as the strongest predictor, followed by Experience, Everyplace, and Exchange. The model demonstrates strong predictive capability, highlighting the importance of behavioral factors such as platform usage, purchase frequency, and social media engagement, while several demographic variables show limited influence. Qualitative findings further support these results, revealing that consumers place strong emphasis on social influence, emotional engagement, and convenience in their online shopping experiences. The study concludes that online cosmetics purchase behavior is shaped by a combination of experiential, relational, and socially driven factors, with social influence playing a dominant role. These findings demonstrate that the 4Es framework remains highly relevant in digitally mediated consumer environments, where purchase decisions are increasingly influenced by interactive experiences, omnichannel accessibility, value co-creation, and consumer advocacy. By integrating quantitative and qualitative evidence, the study extends the application of the 4Es framework beyond traditional marketing contexts and demonstrates its value as a comprehensive model for understanding consumer engagement and online purchasing behavior in contemporary digital marketplaces. The mixed-methods approach provides both generalizable and contextually grounded insights, offering theoretical contributions to digital marketing literature and practical guidance for marketers seeking to strengthen consumer engagement and brand advocacy in increasingly competitive online markets.

## 1. Introduction

The global cosmetics industry has evolved into one of the most dynamic and rapidly expanding sectors within the consumer goods market. Valued at hundreds of billions of dollars annually, the industry encompasses a wide range of products, including skincare, makeup, fragrances, and personal care items that cater to diverse consumer needs and lifestyles. Traditionally driven by innovation, aesthetics, and brand reputation, the cosmetics sector has increasingly embraced digital transformation as consumer behaviors shift toward online platforms and technology-driven engagement. This transformation has been fueled by changing demographics, growing awareness of personal grooming, and the rise in social media influencers who shape consumer perceptions and purchasing decisions (Koetz, 2019; Park & Hong, 2024; Zhang, 2024). In recent years, online cosmetics shopping has emerged as a dominant force reshaping the structure of the beauty market. Digital platforms, as well as e-commerce and s-commerce, have revolutionized how consumers discover, evaluate, and purchase cosmetics products. The convenience of browsing and comparing products, reading reviews, and accessing virtual try-on technologies has empowered consumers to make more informed and personalized decisions. Digital marketing strategies—such as influencer collaborations and live-streaming—have also transformed the online beauty shopping journey from a transactional process into an immersive and interactive experience (Hemachandra & Kusuma, 2024; Jaglan et al., 2024; Shen, 2025).

To understand how businesses can effectively achieve customer engagement and loyalty, scholars and practitioners have increasingly adopted the marketing mix framework as a model suited to the dynamics of the digital era. The traditional 4Ps model (Product, Price, Place, and Promotion) has long guided marketing strategy; however, as consumer behavior becomes increasingly experience-driven and digitally mediated, the 4Es framework (Experience, Exchange, Everyplace, and Evangelism) has emerged as a more holistic approach to understanding customer value creation in the modern marketplace. This model emphasizes emotional engagement, mutual value exchange, ubiquitous accessibility, and the cultivation of brand advocacy. Experience highlights the importance of creating sensory, emotional, and cognitive connections with consumers rather than offering merely functional benefits. Exchange focuses on reciprocal value creation beyond monetary transactions, encompassing trust, transparency, and social interaction. Everyplace underscores the need for seamless accessibility across both physical and digital touchpoints, while Evangelism involves transforming satisfied customers into loyal advocates who voluntarily promote the brand. Collectively, the 4Es framework suggests that consumers no longer respond solely to product-focused strategies; instead, they seek immersive experiences, transparent exchanges, frictionless accessibility, and emotional connections that inspire advocacy (Mada, 2024; Dong & Kongjit, 2025). Furthermore, with the rise in online shopping and social commerce, consumers now interact with brands through digital experiences that shape their trust, satisfaction, and purchase intentions (Dhillon et al., 2022; Singh et al., 2024; Wongsiriwacharakul & Duangekanong, 2024). Studies have examined factors influencing online cosmetics shopping behaviors, such as online service quality, electronic word of mouth (e-WOM), and trust (Golalizadeh et al., 2023; Thanapuech & Pankham, 2024). However, there remains a limited understanding of how consumers perceive their online shopping journeys through an experiential and emotional lens, particularly within the framework of the 4Es.

In Thailand, where the beauty and personal care market continues to expand alongside rapid digital adoption, understanding consumer perceptions of online cosmetic shopping is both timely and essential. Bangkok, as a major hub of digital commerce and lifestyle innovation, provides a dynamic context in which online cosmetics consumption is shaped by cultural nuances, aesthetic preferences, and evolving consumer expectations. Nevertheless, existing research in Thailand has focused primarily on factors such as trust, perceived value, price sensitivity, service quality, and electronic word-of-mouth in online commerce platforms (Padsamritphon & Nagamatsu, 2023; Wongsiriwacharakul & Duangekanong, 2024; Wisedpanich & Wittayakom, 2025). While these studies provide valuable insights into transactional and behavioral determinants of online purchasing, they offer a limited understanding of experiential and relational dimensions, including emotional engagement, immersive consumption experiences, seamless accessibility across digital touchpoints, and consumer advocacy behaviors. Furthermore, these factors are often examined independently rather than through an integrated theoretical framework. Consequently, there remains a need for a more comprehensive approach that captures both the measurable determinants and the interpretive dimensions of consumer behavior. To address this gap, the present study applies the 4Es framework—Experience, Exchange, Everyplace, and Evangelism—to provide a more holistic explanation of online cosmetics purchase behavior in Thailand and to extend understanding of how experiential and advocacy-driven mechanisms influence consumer decision-making in digital marketplaces.

Building on this framework, the present study adopts a mixed-methods design to investigate consumer perceptions of online cosmetics shopping in Bangkok. Specifically, the study pursues three objectives: (1) to assess the individual effects of the 4Es on consumers’ purchase decisions in Thailand’s cosmetics industry; (2) to examine the predictive power of these dimensions in determining the likelihood of online cosmetics purchases; and (3) to identify which of the 4Es exerts the strongest influence on consumer purchase intention within digital marketing contexts. The research question guiding this study is: How do consumers in Bangkok perceive their online cosmetics shopping experiences through the dimensions of the 4Es, and how do these perceptions shape their attitudes and purchase behaviors toward cosmetics brands? The quantitative component evaluates the statistical significance and relative influence of each dimension on purchase decisions, while the qualitative component provides in-depth insights into consumers’ subjective experiences and meaning-making processes. By integrating quantitative and qualitative evidence, this study offers both generalizable and contextually grounded insights. Theoretically, it extends the application of the 4Es framework within a mixed-methods paradigm in an emerging market context. Practically, the findings provide actionable implications for marketers seeking to enhance customer engagement, optimize digital touchpoints, and strengthen brand loyalty in Thailand’s increasingly competitive online beauty market.

## 2. Literature Review

The growth of digital technologies has significantly reshaped consumer behavior in the cosmetics industry, where purchasing decisions are increasingly influenced by online experiences and social interactions. Traditional marketing frameworks are insufficient to explain this shift toward engagement-driven consumption. The 4Es of marketing provide a consumer-centric perspective that emphasizes interaction, accessibility, value, and advocacy in digital environments. Therefore, this literature review synthesizes prior research on the digital transformation of the cosmetics industry, consumer behavior in online environments, the theoretical foundations of the 4Es framework, and the role of demographic and behavioral factors in shaping purchase decisions. By integrating these streams of literature, the study establishes a robust theoretical foundation for examining how the 4Es influence online cosmetics purchase behavior and identifies key research gaps that the present study seeks to address.

### 2.1. Digital Transformation of the Cosmetics Industry

The cosmetics industry has undergone a profound transformation driven by rapid digitalization, reshaping both market structures and consumer engagement processes. Traditionally reliant on physical retail and brand reputation, the industry now operates within a digitally interconnected ecosystem where online platforms, social media, and data-driven marketing play a central role (Park & Hong, 2024; Zhang, 2024). This transformation is not merely technological but strategic, as firms increasingly integrate digital tools to enhance customer experience, personalize offerings, and optimize communication channels (Shen, 2025). Digital marketing innovations—including live-streaming, influencer collaborations, and virtual try-on technologies—have significantly altered how consumers interact with cosmetics brands (Hemachandra & Kusuma, 2024). These tools enable immersive and interactive experiences, shifting the consumer journey from passive consumption to active engagement. Moreover, technological advancements have facilitated real-time feedback loops, allowing brands to respond dynamically to consumer preferences and trends (Sudibyo & Boros, 2024). The rise in social commerce further exemplifies this transformation, where platforms such as TikTok and integrated e-commerce ecosystems combine entertainment with purchasing behavior. As a result, the cosmetics industry has evolved into a highly experiential and digitally mediated environment, where value creation is increasingly driven by emotional engagement, personalization, and community interaction (Koetz, 2019; Park & Hong, 2024; Arbia et al., 2025). Although existing studies consistently acknowledge the growing role of digital technologies in the cosmetics industry, they differ in explaining the primary source of consumer engagement. Some emphasize technological innovation as the key driver of value creation, whereas others highlight social interaction and community participation. This divergence suggests that digital transformation should be viewed as a multidimensional process involving both technological and relational elements. However, the interaction among these elements remains underexplored, particularly in the context of online cosmetics consumption. Therefore, a more integrative approach is needed to explain how experiential, relational, and social factors collectively influence online cosmetics purchasing behavior.

### 2.2. Consumer Behavior in Online Cosmetics Shopping

Consumer behavior in online cosmetics shopping is shaped by a complex interplay of psychological, social, and technological factors. Unlike traditional retail environments, online platforms provide consumers with access to extensive product information, peer reviews, and influencer-generated content, which significantly influence purchase decisions (Jaglan et al., 2024; Singh et al., 2024; Azizah, 2025). Trust has emerged as a critical determinant of online purchasing behavior, particularly in the cosmetics sector, where product authenticity and quality are paramount. Studies indicate that perceived service quality, transparency, and seller credibility significantly influence consumers’ willingness to purchase cosmetics online (Golalizadeh et al., 2023; Thanapuech & Pankham, 2024). Similarly, e-WOM plays a vital role in shaping attitudes and intentions, as consumers rely heavily on reviews and recommendations from other users (Wongsiriwacharakul & Duangekanong, 2024). Social media influencers further amplify these effects by acting as trusted opinion leaders who shape consumer perceptions and preferences. Research demonstrates that both human and virtual influencers can establish emotional connections with consumers, leading to increased engagement and impulsive buying behavior (Hassan et al., 2021; Shamim & Azam, 2024; Yu et al., 2025). These parasocial relationships enhance trust and reduce perceived risk, particularly in online environments where physical product evaluation is not possible. Additionally, demographic and behavioral factors such as age, income, and prior online shopping experience influence purchasing decisions. Younger, digitally literate consumers are more likely to engage in online cosmetics shopping due to their familiarity with digital platforms and openness to new technologies (Ridwan et al., 2025; Wisedpanich & Wittayakom, 2025). Collectively, online cosmetics consumer behavior is characterized by a strong reliance on digital information, social validation, and emotional engagement. Moreover, previous studies have identified numerous determinants of consumer behavior, including trust, electronic word-of-mouth, service quality, and influencer marketing. However, there is limited consensus regarding which of these factors exerts the greatest influence on purchasing decisions. Consequently, this lack of agreement highlights the need for a more integrative perspective that examines multiple dimensions of online consumer engagement simultaneously.

### 2.3. The 4Es of Marketing Framework

The evolution of marketing theory from the traditional 4Ps (Product, Price, Place, Promotion) to more consumer-centric models has led to the emergence of the 4Es framework—Experience, Exchange, Everyplace, and Evangelism (Fei & Kenikasahmanworakhun, 2023; Mada, 2024). This framework reflects the shift from transactional marketing to relational and experiential approaches that emphasize value co-creation and customer engagement. The experience dimension highlights the importance of creating emotional, sensory, and cognitive interactions with consumers. In the cosmetics industry, experiential marketing strategies—such as immersive digital content and storytelling—enhance consumer engagement and brand attachment. These experiences go beyond functional product attributes, focusing instead on emotional satisfaction and identity expression (Koetz, 2019; Dhillon et al., 2022; Songsom, 2026). The exchange dimension redefines value as a reciprocal relationship rather than a simple monetary transaction. It encompasses trust, transparency, and perceived fairness, which are essential in online environments where uncertainty is higher. Consumers increasingly seek authentic interactions and reliable service, reinforcing the importance of relational value (Lee et al., 2025; Songsom, 2026). Furthermore, the everyplace dimension emphasizes seamless accessibility across multiple touchpoints, reflecting the omnichannel nature of modern commerce. Digital platforms enable consumers to interact with brands anytime and anywhere, enhancing convenience and engagement. This ubiquity is particularly relevant in online cosmetics shopping, where consumers rely on integrated digital ecosystems (Park & Hong, 2024; Songsom, 2026). Finally, evangelism refers to the transformation of satisfied customers into brand advocates who actively promote products through social media. Consumer advocacy is driven by positive experiences, trust, and emotional connection, making it a powerful driver of brand growth (Singh et al., 2024; Sharma & Khandeparkar, 2025; Shimul et al., 2026). Overall, the 4Es framework provides a comprehensive lens for understanding contemporary marketing dynamics, particularly in digital environments where consumer engagement is multidimensional and experience-driven. Despite its conceptual relevance, empirical applications of the framework remain relatively limited. Existing studies often focus on individual dimensions rather than examining the combined effects of Experience, Exchange, Everyplace, and Evangelism. Consequently, there is insufficient evidence regarding the relative importance of these dimensions in shaping consumer behavior, particularly within online cosmetics markets.

### 2.4. Demographic Factors in Online Cosmetics Consumption

Demographic variables are fundamental to marketing and consumer behavior research, serving as essential instruments for market segmentation, strategic planning, and target audience identification. In particular, demographic characteristics play a significant role in shaping patterns of online cosmetics consumption (Jothi, 2015; Kalia, 2016; Dhawan & Garga, 2025). Age is one of the most influential factors, with younger consumers demonstrating higher levels of digital engagement and online purchasing behavior due to their familiarity with technology and social media platforms (Abdalla et al., 2025). Generation Z consumers, in particular, exhibit strong preferences for online shopping and are highly influenced by digital content and peer recommendations (Rosnerova et al., 2025; Wisedpanich & Wittayakom, 2025). Gender also plays a critical role in cosmetics consumption, although the gap has narrowed in recent years as both women and an increasing number of men adopt beautification strategies, such as makeup, to enhance their attractiveness. This shift reflects broader societal changes in attitudes toward personal grooming and self-expression (He et al., 2023; Sarpila et al., 2026). Income and education further influence purchasing behavior, as they affect consumers’ access to digital technologies and their ability to evaluate product information. Higher levels of education are associated with greater digital literacy, enabling consumers to navigate online platforms more effectively and make informed decisions. Meanwhile, income levels may determine purchasing frequency, brand preferences, and sensitivity to price and promotions (Rijal et al., 2025). In addition, behavioral factors such as prior online shopping experience and platform usage patterns significantly impact purchase intentions. Consumers who frequently engage in online shopping are more likely to develop trust in digital platforms and exhibit higher purchase intentions (Nasri, 2022; Jiang et al., 2023; Han & Jo, 2025). Although demographic characteristics are widely used for market segmentation, prior findings remain inconsistent regarding their influence on online purchasing behavior. These inconsistencies suggest that demographic factors alone may be insufficient to explain consumer decisions, underscoring the importance of considering behavioral and marketing-related variables in developing a more comprehensive understanding of online cosmetics purchasing behavior.

### 2.5. Hypothesis Development

This study develops hypotheses based on demographic characteristics, behavioral factors, and the 4Es marketing framework to explain online cosmetics purchasing behavior in Thailand. Drawing on the prior literature, the proposed relationships are structured as follows.

Gender differences have long been recognized as an important determinant of consumer behavior, as men and women often differ in their purchasing motivations, information-processing styles, and consumption preferences. In the cosmetics industry, female consumers generally exhibit higher levels of product engagement and are more likely to seek information, evaluate alternatives, and participate in beauty-related consumption activities. These differences may influence purchasing patterns in online environments where product evaluation relies heavily on digital content and social interaction. Prior studies have reported significant gender-related variations in online shopping and cosmetics consumption behavior (He et al., 2023; Rijal et al., 2025; Sarpila et al., 2026). Accordingly, the following hypothesis is proposed:

**H1.**  *Gender has a significant effect on online cosmetics purchase behavior.*

Age influences consumer behavior by shaping lifestyle, technological familiarity, risk perception, and purchasing priorities. Younger consumers are generally more comfortable with digital technologies and social media platforms, making them more receptive to online shopping experiences and digital marketing communications. In contrast, older consumers may place greater emphasis on trust, convenience, and perceived risk when purchasing products online. As online cosmetics purchasing increasingly occurs through digital channels, age-related differences may influence purchasing decisions and engagement levels (He et al., 2023; Hipólito et al., 2025; Sarpila et al., 2026). Therefore, the following hypothesis is proposed:

**H2.**  *Age has a significant effect on online cosmetics purchase behavior.*

Education contributes to consumers’ ability to process information, evaluate alternatives, and make informed purchasing decisions. Individuals with higher educational attainment often possess greater digital literacy and are better equipped to assess product information, online reviews, and promotional claims. Consequently, educational level may influence the way consumers search for information and evaluate cosmetics products in online environments. Previous research has identified education as a relevant factor in shaping online shopping behavior and consumer decision-making (He et al., 2023; Sarpila et al., 2026). Accordingly, the following hypothesis is proposed:

**H3.**  *Educational level has a significant effect on online cosmetics purchase behavior.*

Employment status reflects an individual’s economic circumstances, lifestyle patterns, and daily routines, all of which may influence purchasing behavior. Consumers with stable employment may possess greater financial resources and purchasing power, while differences in work schedules and lifestyle demands can affect shopping preferences and online purchasing frequency. Furthermore, employment status may influence exposure to digital technologies and online retail channels. Prior studies suggest that occupational and employment-related factors can affect consumer purchasing behavior in online contexts (He et al., 2023; Sarpila et al., 2026). Therefore, the following hypothesis is proposed:

**H4.**  *Employment status has a significant effect on online cosmetics purchase behavior.*

Income is a fundamental determinant of purchasing behavior because it directly affects consumers’ ability to allocate resources toward discretionary products such as cosmetics. Higher levels of disposable income generally increase purchasing capacity and expand the range of products consumers are willing to consider. Income may also influence perceptions of product quality, brand preference, and willingness to engage in online shopping. Previous studies have demonstrated that income significantly affects online consumer behavior and purchasing decisions (He et al., 2023; Rijal et al., 2025; Sarpila et al., 2026). Accordingly, the following hypothesis is proposed:

**H5.**  *Monthly income has a significant effect on online cosmetics purchase behavior.*

Social media platforms have become important environments for information exchange, social interaction, and consumer engagement. Through influencer content, peer recommendations, reviews, and user-generated content, social media shapes consumer perceptions and reduces uncertainty during decision-making processes. Different platforms vary in their content formats, levels of interactivity, and mechanisms for social influence, which may affect consumers’ exposure to cosmetics products and purchasing motivations. Prior studies have demonstrated the significant role of social media in influencing online purchase behavior (Pop et al., 2020; Jaglan et al., 2024). Therefore, the following hypothesis is proposed:

**H6.**  *The most frequently used social media platform has a significant effect on online cosmetics purchase behavior.*

Online shopping platforms serve as the primary interface through which consumers search for information, compare products, complete transactions, and interact with sellers. Familiarity with a platform can enhance perceived ease of use, trust, and purchase convenience while reducing uncertainty and transaction costs. Consumers who regularly use particular platforms may therefore develop stronger purchasing habits and greater confidence in online transactions. Previous research highlights the importance of platform characteristics in shaping online purchasing behavior (Nasri, 2022; Kim et al., 2024; Hipólito et al., 2025). Accordingly, the following hypothesis is proposed:

**H7.**  *The most frequently used online shopping platform has a significant effect on online cosmetics purchase behavior.*

Past behavior is often regarded as a strong predictor of future behavior because repeated actions reinforce habits, preferences, and behavioral intentions. Consumers who frequently purchase cosmetics online become more familiar with digital shopping processes and develop greater confidence in evaluating products and completing transactions. As a result, frequent purchasers may exhibit stronger tendencies toward continued online purchasing. Prior research has consistently identified purchase frequency as an important predictor of future purchasing behavior (Nasri, 2022; Kim et al., 2024; Hipólito et al., 2025). Therefore, the following hypothesis is proposed:

**H8.**  *Online cosmetics purchase frequency has a significant effect on online cosmetics purchase behavior.*

Recent purchasing experience reflects consumers’ current engagement with online shopping and may reinforce positive attitudes toward future purchases. Consumers who have recently purchased cosmetics online are likely to possess stronger product familiarity, greater confidence in digital transactions, and more favorable perceptions of online shopping. Such experiences can reduce uncertainty and strengthen purchase intentions. Previous studies indicate that recent purchasing behavior is closely associated with future purchasing decisions (Nasri, 2022; Kim et al., 2024; Hipólito et al., 2025). Accordingly, the following hypothesis is proposed:

**H9.**  *Recent online cosmetics purchase has a significant effect on online cosmetics purchase behavior.*

Experience emphasizes the creation of memorable emotional, sensory, and cognitive interactions between consumers and brands. Experiential marketing theory suggests that positive experiences enhance satisfaction, engagement, and emotional attachment, which in turn influence purchasing decisions. In online cosmetics shopping, immersive content, visual presentation, interactive features, and personalized experiences may increase consumers’ willingness to purchase products. Previous research supports the importance of experiential factors in shaping consumer behavior (Koetz, 2019; Dhillon et al., 2022; Songsom, 2026). Therefore, the following hypothesis is proposed:

**H10.**  *Experience has a significant effect on online cosmetics purchase behavior.*

The Everyplace dimension reflects consumers’ ability to access information, products, and services seamlessly across multiple digital touchpoints. Omnichannel accessibility enhances convenience, reduces effort, and enables consumers to interact with brands whenever and wherever they choose. By increasing accessibility and reducing barriers to purchase, Everyplace can strengthen consumer engagement and facilitate purchasing behavior. Prior studies have highlighted the importance of accessibility and omnichannel integration in digital commerce (Mada, 2024; Park & Hong, 2024; Songsom, 2026). Accordingly, the following hypothesis is proposed:

**H11.**  *Everyplace has a significant effect on online cosmetics purchase behavior.*

Exchange focuses on the value consumers receive relative to the costs incurred during a transaction. Beyond monetary considerations, consumers evaluate value through perceptions of quality, fairness, trust, transparency, and service reliability. When consumers perceive that online transactions provide favorable value and reduce risk, they are more likely to proceed with purchases. Previous research has demonstrated the importance of perceived value and trust in influencing online consumer behavior (Golalizadeh et al., 2023; Thanapuech & Pankham, 2024; Lee et al., 2025; Songsom, 2026). Therefore, the following hypothesis is proposed:

**H12.**  *Exchange has a significant effect on online cosmetics purchase behavior.*

Evangelism represents consumers’ willingness to advocate for brands and share experiences with others through reviews, recommendations, and word-of-mouth communication. From a social influence perspective, advocacy behaviors reduce perceived risk, enhance credibility, and provide social validation for potential buyers. Because cosmetics products are often evaluated through subjective experiences and peer recommendations, consumer advocacy can exert a particularly strong influence on purchasing decisions. Prior studies emphasize the importance of e-WOM, brand advocacy, and social influence in digital marketplaces (Wongsiriwacharakul & Duangekanong, 2024; Sharma & Khandeparkar, 2025; Shimul et al., 2026). Accordingly, the following hypothesis is proposed:

**H13.**  *Evangelism has a significant effect on online cosmetics purchase behavior.*

Collectively, these hypotheses reflect the view that online cosmetics purchase behavior is shaped by a combination of demographic characteristics, behavioral experiences, and marketing-related influences. While demographic factors provide important contextual insights, the 4Es framework offers a more comprehensive understanding of how experiential engagement, accessibility, perceived value, and consumer advocacy influence purchasing decisions in digital environments. Accordingly, the proposed hypotheses are empirically tested to evaluate the relative effects of these factors on online cosmetics purchase behavior in Thailand.

## 3. Materials and Methods

This study employed a sequential explanatory mixed-methods design, in which quantitative data collection and analysis were conducted first, followed by a qualitative phase to further explain and contextualize the findings. This approach was appropriate because the study sought to examine both the measurable relationships between the 4Es dimensions and online cosmetics purchase behavior and the underlying meanings, perceptions, and experiences that shape consumer decision-making. While the quantitative phase identified the significance and relative influence of the proposed variables, it provided limited insight into the subjective experiences and social processes underlying these relationships. The subsequent qualitative phase therefore enabled a deeper exploration of consumer perspectives, enriching the interpretation of the statistical results and providing a more comprehensive understanding of how and why the 4Es influence online cosmetics purchasing behavior in Thailand. By integrating quantitative and qualitative evidence, the study enhances both the comprehensiveness and explanatory power of its findings.

### 3.1. Quantitative Approach

The quantitative component of this study was conducted using a structured online questionnaire administered via Google Forms. Measurement items were developed based on an extensive review of prior literature on online consumer behavior, digital marketing, and the 4Es framework, and were adapted from established and validated scales. The instrument was designed to measure the four key constructs of the 4Es model alongside demographic and behavioral variables relevant to online cosmetics consumption. All latent constructs were operationalized using multi-item Likert-scale measures ranging from 1 (strongly disagree) to 5 (strongly agree). Experience captured consumers’ emotional and sensory engagement during online cosmetics shopping; Exchange measured perceived value, trust, and fairness in transactions; Everyplace assessed accessibility and convenience across digital platforms; and Evangelism reflected consumers’ willingness to recommend and advocate for cosmetics brands. The dependent variable, online cosmetics purchase behavior, was measured based on respondents’ self-reported purchasing decisions. Demographic variables and behavioral factors were included as control and explanatory variables.

To ensure content validity, the questionnaire was reviewed by three experts in marketing, digital commerce, and research methodology. The Item Objective Congruence (IOC) values ranged from 0.80 to 1.00, indicating strong alignment between the measurement items and research objectives. A pilot test involving 30 participants was conducted to evaluate clarity, reliability, and instrument performance. Based on the pilot results, minor revisions were made to improve wording, response structure, and logical flow. Construct reliability and validity were assessed prior to hypothesis testing. Internal consistency reliability was assessed using Cronbach’s alpha (α = 0.79), indicating that all constructs exceeded the recommended threshold of 0.70 and demonstrated acceptable reliability. Composite reliability (CR) values were also above 0.70, confirming satisfactory reliability. Convergent validity was established through average variance extracted (AVE), with all values exceeding the 0.50 criterion. Discriminant validity was assessed using the Fornell–Larcker criterion, ensuring that the square root of each construct’s AVE exceeded its correlations with other constructs. The finalized questionnaire was distributed between November and December 2025 through multiple online platforms, including Facebook and LINE, to maximize reach and accessibility among the target population.

The target population consisted of individuals residing in Thailand, aged 18 years and above, with prior experience in online cosmetics shopping. The required sample size was determined using Cochran’s formula at a 95% confidence level and a 5% margin of error, resulting in a minimum requirement of 384 respondents (Uakarn et al., 2021). To enhance statistical power and robustness, a total of 1000 valid responses were collected using convenience sampling, thereby exceeding the recommended threshold.

Data analysis was conducted using Jamovi (version 2.16.17.0). Descriptive statistics were used to summarize respondents’ characteristics, while binary logistic regression was employed to examine the effects of the 4Es dimensions, demographic variables, and behavioral factors on online cosmetics purchase behavior among Thai consumers. This technique was selected because the dependent variable was dichotomous, indicating whether respondents engaged in online cosmetics purchasing. It is appropriate for estimating the probability of a binary outcome, assessing the relative contribution of multiple predictors, and generating odds ratios that indicate the strength and direction of relationships between independent variables and purchase behavior.

Prior to analysis, all variables were coded appropriately. The dependent variable was coded as 1 = online cosmetics purchaser and 0 = non-purchaser. Gender (0 = female, 1 = male) and recent online cosmetics purchase (0 = no, 1 = yes) were coded as binary variables. Age, educational level, employment status, income, most frequently used social media platform, most frequently used online shopping platform, and online cosmetics purchase frequency were coded according to their ordered response categories, with the lowest category serving as the reference group where applicable. The four dimensions of the 4Es framework—Experience, Exchange, Everyplace, and Evangelism—were operationalized as continuous variables using composite scores calculated from their respective Likert-scale measures. Model performance was evaluated using the Omnibus Test of Model Coefficients, Nagelkerke R^2^, and classification accuracy. These indicators were used to assess the explanatory power, overall model fit, and predictive performance of the binary logistic regression model. This analytical approach enabled the identification of significant predictors and the assessment of their relative influence on consumers’ purchasing decisions in Thailand.

### 3.2. Qualitative Approach

To complement the quantitative findings and provide deeper insights into consumers’ perceptions, a qualitative phase was conducted using in-depth semi-structured interviews. This phase aimed to further explain and contextualize the statistical relationships identified in the quantitative analysis, particularly regarding how the 4Es dimensions shape online cosmetics purchasing behavior. The qualitative component adhered to established reporting standards, specifically the COREQ checklist, to ensure methodological rigor, transparency, and credibility.

From a research design perspective, the qualitative phase adopted a pragmatic interpretivist approach, focusing on how consumers construct and interpret their online cosmetics shopping experiences within digitally mediated environments. Semi-structured interviews were selected as the primary data collection method due to their flexibility in capturing rich, detailed narratives while maintaining consistency across key themes derived from the quantitative results and literature review. The interview guide was systematically developed based on the 4Es framework and refined through expert consultation in marketing and consumer behavior. The questions were structured to progress from general online shopping experiences to more specific dimensions, including emotional engagement (experience), perceived value and trust (exchange), accessibility and convenience (everyplace), and advocacy behaviors (evangelism).

Regarding researcher involvement and reflexivity, the interviews were conducted by the research team, who possess formal training in qualitative methods and expertise in digital marketing and consumer behavior. No prior relationships existed between the researchers and participants, thereby reducing potential bias. Participants were informed of the study’s objectives and the researchers’ academic backgrounds prior to the interviews. Reflexive memoing was undertaken throughout the research process to critically examine potential assumptions, particularly those related to digital consumption behavior and online cosmetics purchasing.

For participant selection, purposive sampling was initially employed to recruit individuals who had prior experience with online cosmetics shopping in Thailand. The inclusion criteria required participants to: (1) be Thai residents; (2) be at least 18 years old; and (3) have recent experience purchasing cosmetics through online platforms. To enhance the diversity of perspectives, participants were selected across different demographic backgrounds and levels of online shopping experience. Purposive sampling was subsequently applied to identify additional participants with relevant experiences. In total, 12 participants were interviewed, which is consistent with qualitative research standards suggesting that a sample of 6–10 participants is sufficient to achieve data saturation in focused studies (Sebele-Mpofu, 2020). Data collection was conducted in January 2026, with interviews carried out either face-to-face or via online communication platforms, depending on participant preference. Each interview lasted approximately 45–60 min and was conducted in Thai. With participants’ consent, all interviews were audio-recorded and transcribed verbatim. Field notes were also maintained to capture contextual details and preliminary analytical insights.

The data were analyzed using qualitative content analysis through a systematic multi-step process. Initially, transcripts were reviewed repeatedly to achieve familiarity with the data. Open coding was then conducted to identify meaningful units related to consumer perceptions of online cosmetics shopping, particularly across the 4Es dimensions. These codes were subsequently grouped into broader categories, which were further refined into overarching themes through iterative comparison. The final themes reflected key aspects of consumer experience, perceived value and trust, platform accessibility, and advocacy behaviors. To ensure analytical rigor, the findings were continuously compared with the original data for consistency and coherence.

To enhance trustworthiness, several strategies were employed, including reflexive memoing, peer debriefing among the research team, and the maintenance of a transparent audit trail documenting coding and analytical decisions. Selected participants were also invited to review summary interpretations to ensure accuracy and alignment with their perspectives. Through this rigorous qualitative process, the findings provided rich contextual explanations that complemented and extended the quantitative results, thereby strengthening the overall validity and depth of the study.

A word cloud visualization was generated from the transcribed interview data. The visualization served as an exploratory tool to identify frequently occurring words and recurring expressions in participants’ narratives. By highlighting dominant terms, the word cloud provides an overview of key lexical patterns associated with core dimensions of online cosmetics consumption. Although the word cloud does not function as a primary analytical technique, it complements the analysis by reinforcing the salience of recurring concepts and supporting the interpretation of patterns identified during the coding process.

## 4. Results

Drawing on data from a structured survey, this study examines the factors influencing online cosmetics purchase behavior through the lens of the 4Es framework, with in-depth interviews providing deeper insights into how consumers in Thailand perceive and experience digital cosmetics shopping. The quantitative findings identify key demographic, behavioral, and marketing-related determinants, whereas the qualitative results capture the underlying meanings, emotions, and social influences shaping consumer decisions. Collectively, these findings offer a comprehensive understanding of how experiential, relational, and socially driven factors interact to influence online cosmetics purchasing behavior in the Thai digital marketplace.

### 4.1. Quantitative Results

The study’s respondents were selected using convenience sampling to ensure a diverse demographic representation aligned with the study’s objectives. This approach enabled efficient data collection and supported robust quantitative analysis. Following data collection, a rigorous data cleansing process was conducted, including the removal of incomplete responses, correction of errors, and exclusion of outliers, thereby enhancing data accuracy and reliability. The resulting dataset provides a sound basis for identifying general trends and supporting meaningful interpretation of consumer behavior. Table 1 summarizes the final sample characteristics.

Table 1 summarizes the demographic and behavioral characteristics of 1000 respondents, indicating a relatively balanced gender distribution and a predominance of young adults, particularly those aged 20–30 years. Most respondents hold at least a bachelor’s degree and are single, reflecting a digitally literate consumer group. Income levels are primarily within the lower-to-middle-income range, suggesting broad accessibility of online cosmetics. In terms of digital behavior, TikTok and Shopee emerge as dominant platforms, highlighting the role of social commerce. Respondents report frequent online cosmetics purchases, with a majority having made recent purchases and expressing strong future purchase intentions, indicating a high level of engagement in online cosmetics consumption in Thailand.

Table 2 presents the descriptive statistics of the study variables, indicating moderate variation across demographic characteristics and active engagement in online cosmetics purchasing. Respondents report frequent purchases, high recent buying activity, and strong future purchase intentions. The 4Es dimensions—Experience, Exchange, Everyplace, and Evangelism—exhibit relatively high mean scores with low variability, reflecting generally positive and consistent consumer perceptions. Among these, Evangelism records the highest mean, highlighting the importance of peer influence and word-of-mouth. Overall, the results suggest strong consumer engagement and provide a reliable basis for subsequent inferential analysis.

Table 3 presents the Omnibus Test of Model Coefficients, indicating that the logistic regression model is statistically significant. This result confirms that the set of independent variables significantly improves model fit compared to the null model, demonstrating their collective ability to explain variations in online cosmetics purchase behavior. The findings support the adequacy of the model and justify further analysis of individual predictors.

Table 4 presents the model summary of the binary logistic regression, indicating good model fit and strong explanatory power. The −2 Log Likelihood reflects an improved fit compared to the null model, while the model converged after six iterations, confirming estimation stability. The Cox and Snell R^2^ and Nagelkerke R^2^ values suggest that the model explains a substantial proportion of variance in online cosmetics purchase behavior. The results demonstrate that the model has strong predictive capability in explaining consumer decisions in the digital cosmetics context.

Table 5 presents the classification results of the logistic regression model, demonstrating strong predictive accuracy. These findings indicate that the model effectively distinguishes between consumers who do and do not purchase cosmetics online. The balanced classification performance across both groups further confirms the robustness and practical relevance of the model, supporting its suitability for predicting online cosmetics purchase behavior in Thailand.

The predictive regression equation of Model 1 using the coefficients from Table 6 can be described by the following equation:

(1) $$P=\frac{1}{1+e^{-z}}$$

where *P* is online cosmetics purchasing in Thailand, and *Z* = −13.288 − 0.505(Gender) − 0.946(Income) + 0.110(Most frequently used social media platform) + 0.890(Most frequently used online shopping platform) + 0.675(Online cosmetics purchase frequency) + 0.654(Recent online cosmetics purchase) + 0.804(Experience) + 0.326(Everyplace) + 0.161(Exchange) + 1.740(Evangelism).

Table 6 reports the binary logistic regression results, providing insight into how each variable influences the likelihood of online cosmetics purchasing in Thailand. The findings support 10 of the 13 hypotheses, with H1 and H5–H13 accepted and H2–H4 rejected. Among the demographic variables, only gender and income demonstrate significant effects, whereas age, education, and employment status do not contribute meaningfully when other factors are considered simultaneously. This finding suggests that online cosmetics purchasing is shaped less by traditional demographic segmentation and more by consumers’ engagement with digital platforms and shopping activities.

Behavioral variables play an important role in explaining purchase behavior. Consumers who frequently purchase cosmetics online, have recent purchasing experience, and regularly use online shopping platforms are more likely to engage in future online cosmetics purchases. These findings highlight the importance of familiarity, habitual behavior, and continued interaction with digital shopping environments in reinforcing consumer purchasing decisions.

The results also provide strong support for the 4Es framework. All four dimensions demonstrate positive relationships with online cosmetics purchasing behavior, indicating that consumer decisions are influenced by experiential, relational, accessibility-related, and advocacy-driven factors. Among these dimensions, Evangelism emerges as the most influential factor, suggesting that recommendations, reviews, and consumer advocacy play a particularly important role in shaping purchasing decisions. Experience represents the second strongest influence, emphasizing the value of emotionally engaging and immersive shopping experiences. Everyplace and Exchange also contribute positively, highlighting the importance of seamless accessibility and perceived value in encouraging online cosmetics purchases.

Collectively, the findings suggest that successful digital cosmetics marketing strategies should extend beyond product-focused approaches and prioritize consumer engagement, platform accessibility, and brand advocacy. The prominence of Evangelism further indicates that social influence and community-driven interactions have become central drivers of purchasing behavior in contemporary digital marketplaces.

### 4.2. Qualitative Results

The qualitative findings complement the quantitative results by providing contextual and analytical insights. Participant profiles (Table 7) are presented to contextualize the data, followed by a content analysis of the 4Es in shaping consumer perceptions and online cosmetics purchase decisions. This approach ensures that the findings are grounded in lived experience, enhancing their credibility and practical relevance in the Thai digital cosmetics context.

Table 7 presents the demographic and behavioral profiles of 12 interview participants, reflecting a diverse sample in terms of gender, age (20–42 years), and occupational backgrounds. Respondents include students and professionals from various fields, offering multiple perspectives on online cosmetics consumption. Shopee, Lazada, and TikTok are identified as the dominant platforms, while purchases span a wide range of beauty products, including skincare, makeup, and haircare. The diversity of the sample enhances the richness and depth of the qualitative analysis and supports a comprehensive understanding of consumer experiences within the 4Es framework.

**Figure 1:**
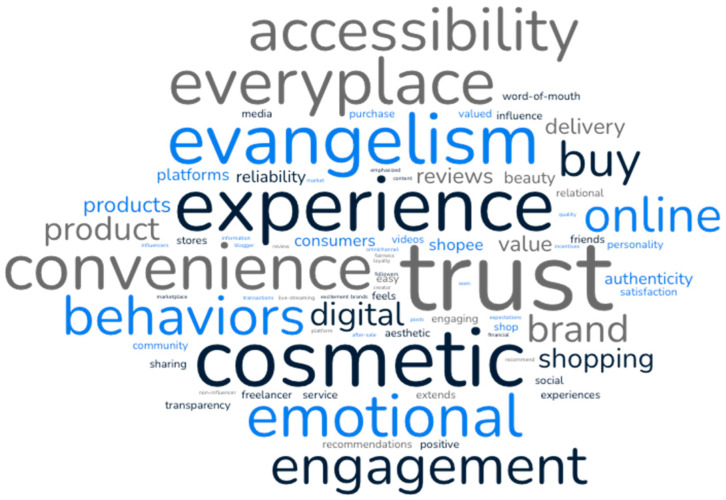
Word cloud of key themes in online cosmetics shopping experiences based on qualitative interview data.

A word cloud visualization was generated from the interview data to provide an initial overview of dominant themes in participants’ narratives. Figure 1 illustrates the most frequently occurring terms related to online cosmetics shopping, offering a visual representation of key concepts prior to in-depth thematic interpretation.

Figure 1 presents a word cloud generated from the qualitative interview data, offering a visual summary of the most frequently occurring terms in participants’ narratives regarding online cosmetics shopping. The prominence of keywords such as trust, experience, evangelism, convenience, and engagement indicates the central themes shaping consumer perceptions and behaviors. Notably, *trust* and *experience* emerge as dominant concepts, highlighting the importance of reliable platforms and positive user interactions in influencing purchase decisions. Terms such as *everyplace* and *accessibility* further emphasize the role of convenience and seamless access in digital environments, while *evangelism*, *reviews*, and *influence* reflect the significant impact of word-of-mouth and social validation. Additionally, the presence of words like *emotional*, *brand*, and *value* suggests that both affective and cognitive factors contribute to consumer decision-making. The visualization reinforces the relevance of the 4Es framework—Experience, Exchange, Everyplace, and Evangelism—by illustrating how these dimensions are embedded in consumers’ lived experiences, thereby supporting the thematic findings of the qualitative analysis.

#### 4.2.1. Experience: Emotional and Sensory Engagement in the Digital Environment

Participants described online cosmetics shopping as a highly immersive and emotional process rather than a mere act of consumption. Several respondents noted that browsing through digital storefronts, watching influencers’ product demonstrations, and engaging with aesthetic visuals created a sense of excitement and curiosity. R1, a university student, remarked, “*When I scroll through TikTok, the videos are so visually pleasing—it feels like I’m already part of the brand before I even buy anything*” (personal communication, 13 January 2026). Similarly, R6, an office employee, explained that “*When I watch influencer demonstrations, see attractive product visuals, or try interactive features online, I can picture how the products would look on me and how they fit the image I want to project. It makes me feel more connected to the brand, more confident in my choice, and more excited about the purchase*” (personal communication, 17 January 2026).

Furthermore, the sensory appeal of product imagery, combined with interactive features such as live-streaming and virtual try-ons, enhanced the experiential value of online shopping. Participants also highlighted emotional connection and self-expression as key motivators. R8, another student, shared, “*I enjoy choosing colors and styles that match my personality. Online stores make it easy to explore new looks and trends*” (personal communication, 18 January 2026). The findings indicate that digital experience in cosmetics shopping transcends functionality, as it allows consumers to express identity, creativity, and self-confidence through aesthetic discovery. This dimension of Experience thus represents the emotional heart of the consumer journey, where pleasure, beauty, and technology converge.

Beyond the immediate shopping experience, these findings suggest that consumers increasingly view digital cosmetics platforms as spaces for identity construction and self-presentation. Emotional engagement appears to arise not only from product evaluation but also from the symbolic meanings associated with beauty, lifestyle, and personal expression. Participants frequently described how visual content, influencer demonstrations, and interactive features enabled them to imagine desired versions of themselves and connect cosmetics products with personal aspirations. In addition, social validation through reviews, influencer endorsements, and online communities helped consumers interpret product value, reduce uncertainty, and gain confidence in their purchasing decisions. These findings indicate that digitally mediated experiences shape consumer behavior by combining emotional engagement with social meaning-making, helping explain why Experience emerged as a significant predictor of online cosmetics purchasing behavior in the quantitative analysis.

#### 4.2.2. Exchange: Trust, Transparency, and Perceived Value

The second major theme centers on Exchange, which reflects consumers’ perceptions of fairness, reliability, and value in online transactions. Respondents frequently emphasized that mutual trust and transparent communication are vital in influencing purchase decisions. R7, a government officer, stated, “*I always check for real reviews and seller ratings before buying. If the shop hides information or doesn’t reply to questions, I won’t trust them*” (personal communication, 17 January 2026). Similarly, R10, a sales manager, noted that “*Authenticity and after-sale service matter more than discounts. I’d rather pay a little more for a brand I can trust*” (personal communication, 21 January 2026).

Promotional deals and free samples were perceived as valuable, yet participants clarified that long-term loyalty stemmed from consistent product quality and customer care. R2, an office employee, described his experience with Lazada: “*When the seller included a small gift and followed up after delivery, I felt valued. It made me want to buy it again*” (personal communication, 13 January 2026). Respondents also mentioned that platforms offering easy refund policies, reliable logistics, and clear descriptions fostered greater confidence. This finding underscores that Exchange extends beyond economic transaction—it represents a relational bond between brand and buyer built on reciprocity, authenticity, and shared value.

These findings further suggest that Exchange operates as a risk-reduction mechanism within digital environments where consumers cannot physically inspect products before purchase. Rather than focusing exclusively on price considerations, participants evaluated value through signals of authenticity, responsiveness, and reliability. This reflects a shift from transactional exchange toward relational exchange, where trust becomes a strategic resource that reduces uncertainty and strengthens long-term customer relationships. The findings therefore indicate that perceived value is co-created through ongoing interactions between consumers, brands, and platforms, highlighting the importance of transparency and credibility in shaping online purchasing decisions.

#### 4.2.3. Everyplace: Seamless Accessibility and Platform Convenience

The Everyplace dimension reflects consumers’ strong appreciation for the accessibility, speed, and convenience of digital platforms. Participants consistently highlighted that online cosmetics shopping fits seamlessly into their busy lifestyles, allowing them to shop “anytime and anywhere” with minimal effort. For instance, R4, a freelancer, commented, “*I like that I can buy skincare while waiting for clients or during my lunch break. It saves time and effort*” (personal communication, 15 January 2026). Shopee and Lazada were praised for their structured interfaces, ease of navigation, and reliable delivery systems, while TikTok was perceived as more engaging, interactive, and discovery-oriented, particularly for exploring new products.

Respondents valued the integration of multiple features—search filters, personalized recommendations, secure payment methods, and fast delivery. R11, a female freelancer, expressed, “*I often find new products from TikTok videos, but I order from Shopee because I trust their delivery system more*” (personal communication, 22 January 2026). However, concerns were raised about the authenticity of products sold via social media. R3, a service worker, cautioned, “*Sometimes, I see good deals on TikTok but I’m not sure if they’re real products. I prefer official stores on their platforms*” (personal communication, 14 January 2026). These findings illustrate how Everyplace reflects consumers’ expectations for omnichannel connectivity, reliability, and convenience—key factors that influence satisfaction and continued engagement in the online cosmetics marketplace.

The findings suggest that Everyplace extends beyond simple convenience by influencing consumers’ perceptions of control and efficiency throughout the purchasing journey. The ability to seamlessly move across platforms, compare products, access information, and complete transactions creates a frictionless consumer experience that encourages continued engagement. Furthermore, participants’ preference for platforms that combine discovery, evaluation, and purchasing functions illustrates the growing importance of ecosystem integration in digital commerce. This indicates that accessibility serves not merely as a functional attribute but as a strategic factor that enhances consumer confidence, satisfaction, and purchasing continuity.

#### 4.2.4. Evangelism: Word-of-Mouth, Advocacy, and Digital Community Influence

The final theme, Evangelism, captures how satisfied consumers become voluntary brand advocates who spread positive word-of-mouth both online and offline. Many participants reported sharing their shopping experiences through social media posts, reviews, and recommendations to friends. For instance, R5, a 30-year-old beauty blogger, shared, “*When I find a product that really works, I post about it right away. My followers trust my reviews, and I feel proud to recommend good brands*” (personal communication, 16 January 2026). Similarly, R9, a content creator, said, “*I don’t advertise every brand—only those that match my personality and values. People can tell when your reviews are honest*” (personal communication, 20 January 2026).

Even non-influencer participants demonstrated advocacy behaviors through casual digital sharing. R12, an office employee, explained, “*If I like a product, I’ll tell my friends or post a short review on Shopee. It feels good to help others find something useful*” (personal communication, 22 January 2026). This indicates that evangelism is not limited to formal influencers but extends to everyday consumers who build community trust and shape collective brand perception. Positive experiences inspired by authenticity, transparency, and emotional resonance naturally evolved into advocacy, suggesting that brand evangelism in Thailand’s online beauty market is driven by relational satisfaction and shared values rather than financial incentives.

These findings further suggest that evangelism reflects a broader process of social value creation within digital communities. Consumers who share experiences and recommendations are not only transmitting product information but also contributing to collective trust and community knowledge. Advocacy behaviors appear to be motivated by a desire for social connection, credibility, and value alignment rather than solely by satisfaction with product performance. This helps explain why Evangelism emerged as the strongest predictor in quantitative analysis. In highly connected digital environments, consumers increasingly rely on peer-generated information and community validation, making advocacy a powerful mechanism through which purchasing decisions are shaped and reinforced.

## 5. Discussion

This study provides robust empirical support for the applicability of the 4Es framework (Experience, Exchange, Everyplace, and Evangelism) in explaining online cosmetics purchase behavior in Thailand, thereby addressing all three research objectives and the guiding research question. The findings not only confirm the significance of the 4Es dimensions but also extend prior research by integrating quantitative and qualitative evidence within a mixed-methods design.

In relation to the predictive power of the model, the results demonstrate strong explanatory capability, with a Nagelkerke R^2^ of 0.574 and an overall classification accuracy of 84.5%. These findings indicate that the integration of the 4Es with demographic and behavioral variables provides a comprehensive model for predicting online cosmetics purchase behavior. This supports prior research suggesting that consumer behavior in digital environments is shaped by a combination of psychological, technological, and behavioral factors (Chang et al., 2016; Azizah, 2025; Songsom, 2026).

Regarding demographic and behavioral variables, gender (H1) and income (H5) are statistically significant, supporting prior research that highlights differences in cosmetics consumption patterns and purchasing power across demographic groups (He et al., 2023; Rijal et al., 2025; Sarpila et al., 2026). In contrast, age (H2), education (H3), and employment status (H4) are not statistically significant, suggesting that traditional demographic segmentation may be less relevant in digitally mature markets. Meanwhile, behavioral variables—including online shopping platform usage (H7), purchase frequency (H8), and recent purchase experience (H9)—are all significant predictors, confirming that familiarity, habitual behavior, and past experiences enhance consumer confidence and increase purchase likelihood (Nasri, 2022; Kim et al., 2024; Hipólito et al., 2025). The significance of social media platform usage (H6) aligns with prior research demonstrating the central role of social commerce and influencer-driven engagement in shaping purchase intentions (Pop et al., 2020; Jaglan et al., 2024).

With regard to the individual effects of the 4Es, the quantitative results indicate that all four dimensions significantly influence online cosmetics purchase behavior, supporting H10–H13. Among these, Evangelism emerges as the most influential factor, followed by Experience, Everyplace, and Exchange. This finding aligns with prior literature emphasizing the role of e-WOM, influencer marketing, and brand advocacy in shaping consumer decisions in digital environments. Consumer-generated content and peer recommendations significantly enhance trust and purchase intention, particularly in the cosmetics sector, where product evaluation is highly subjective and experiential (Wongsiriwacharakul & Duangekanong, 2024; Sharma & Khandeparkar, 2025; Shimul et al., 2026). The prominence of Evangelism underscores the pivotal role of social influence, suggesting that modern consumers rely heavily on social validation and community engagement when making purchasing decisions. This extends prior research on brand advocacy, indicating that satisfied customers who actively promote products contribute significantly to brand growth and consumer trust (Sharma & Khandeparkar, 2025; Shimul et al., 2026). The study’s strong effect of Evangelism confirms that advocacy-driven mechanisms, such as influencer endorsements and online reviews, are central to consumer decision-making in the online cosmetics market.

Similarly, the significant positive effect of Experience supports previous research highlighting the importance of emotional and sensory engagement in digital marketing. Experiential marketing strategies—such as immersive content, storytelling, and influencer demonstrations—have been found to enhance consumer satisfaction and strengthen brand attachment (Koetz, 2019; Dhillon et al., 2022; Songsom, 2026). The present findings confirm that online cosmetics shopping is not merely transactional but deeply experiential, where aesthetic appeal and emotional resonance play a critical role in influencing purchase behavior.

Furthermore, the Everyplace dimension demonstrates a significant positive effect, consistent with prior studies emphasizing the importance of accessibility, convenience, and omnichannel integration in digital commerce (Mada, 2024; Park & Hong, 2024; Songsom, 2026). Consumers increasingly expect seamless interactions across multiple platforms, enabling them to browse, evaluate, and purchase products anytime and anywhere. Consequently, digital accessibility enhances engagement and reduces friction throughout the consumer journey.

While Exchange is statistically significant, its relatively weaker effect suggests that perceived value, trust, and fairness function as necessary but not sufficient conditions for purchase decisions. The study’s finding is consistent with prior studies that highlight the importance of trust and service quality in reducing perceived risk in online transactions (Golalizadeh et al., 2023; Thanapuech & Pankham, 2024; Lee et al., 2025; Songsom, 2026). However, in highly competitive and experience-driven markets such as cosmetics, these factors may primarily serve as baseline expectations rather than key differentiators that actively drive consumer choice.

Importantly, the qualitative findings provide rich contextual insights that complement and reinforce the quantitative results. Participants’ narratives strongly support the importance of Evangelism, the strongest predictor in the quantitative analysis, as many respondents described actively sharing product experiences, posting reviews, and influencing others’ decisions. This aligns with prior studies indicating that e-WOM and social media interactions play a crucial role in shaping consumer attitudes and behaviors (Pop et al., 2020; Wongsiriwacharakul & Duangekanong, 2024). More importantly, the findings suggest that consumers function not only as purchasers but also as active contributors to brand meaning and credibility within digital communities. Through reviews, recommendations, and social sharing, consumers participate in the co-creation of trust, thereby amplifying brand influence beyond firm-controlled marketing communications. This helps explain why Evangelism emerged as the strongest predictor in the quantitative analysis, as purchasing decisions were strongly influenced by peer-generated information, community validation, and consumer advocacy rather than solely by brand-generated messages.

The qualitative findings also deepen the understanding of Experience, revealing that consumers perceive online cosmetics shopping as an emotionally engaging and aesthetically driven process. Participants emphasized the role of visual content, influencer demonstrations, and self-expression, which supports existing research on experiential marketing and digital engagement (Koetz, 2019; Songsom, 2026). These findings suggest that experiential value extends beyond product evaluation and serves as a mechanism through which consumers connect products with desired identities, lifestyles, and emotional aspirations. Through interactions with digital content, consumers actively construct and express personal identities, transforming cosmetics shopping from a purely transactional activity into a symbolic and emotionally meaningful experience. Furthermore, social validation emerged as a key mechanism linking emotional engagement to purchase behavior, as consumers frequently relied on peer reviews and influencer endorsements to reduce uncertainty and validate their product choices (Utami et al., 2025; Rukhayati & Ali, 2026). This indicates that online cosmetics purchasing is shaped not only by functional evaluations of product attributes but also by socially mediated interpretations of value, trust, and self-expression. Collectively, these insights help explain why Experience emerged as one of the strongest predictors in quantitative analysis.

Furthermore, the qualitative data enrich the interpretation of Exchange by highlighting that trust is built through transparency, authenticity, and consistent service rather than price alone. This supports prior findings that emphasize the importance of trust and perceived fairness in online transactions (Golalizadeh et al., 2023; Lee et al., 2025). The findings further suggest that trust functions as a risk-reduction mechanism in online cosmetics purchasing, where consumers cannot physically evaluate products prior to purchase. Consequently, transparent communication and reliable service become critical signals that reduce uncertainty and strengthen purchase confidence.

Similarly, the qualitative data illustrate the Everyplace dimension through participants’ emphasis on convenience and platform integration, confirming previous research on the role of accessibility in enhancing consumer engagement (Park & Hong, 2024; Shen, 2025). Beyond convenience, the findings indicate that seamless accessibility influences consumers’ perceptions of efficiency, control, and ease of decision-making throughout the purchasing journey. Participants frequently described the ability to search for products, compare alternatives, access information, and complete purchases across multiple platforms with minimal effort. This suggests that omnichannel accessibility serves not merely as a functional benefit but also as a strategic driver of continued engagement and purchasing behavior.

Taken together, the integration of quantitative and qualitative findings provides a comprehensive answer to the research question. Consumers in Bangkok perceive online cosmetics shopping as a multidimensional experience shaped by emotional engagement (Experience), relational trust (Exchange), seamless accessibility (Everyplace), and social influence (Evangelism). Among these, Evangelism exerts the strongest influence, reflecting the increasing importance of community-driven and socially embedded consumption in digital environments.

### 5.1. Research Contributions

This study makes several theoretical contributions to the digital marketing and consumer behavior literature. First, the findings both confirm and extend existing knowledge regarding the 4Es framework. Consistent with prior studies, all four dimensions of Experience, Exchange, Everyplace, and Evangelism significantly influence consumer behavior in digital environments, supporting the continued relevance of engagement-based marketing frameworks. However, the present study extends existing theory by demonstrating a clear hierarchy among these dimensions within the context of online cosmetics purchasing in an emerging market. Specifically, Evangelism emerged as the strongest predictor of purchase behavior, suggesting that consumer advocacy and peer-generated influence may exert greater effects on purchasing decisions than experiential or transactional factors in social-commerce environments.

Second, the findings contribute to the growing literature on social commerce by illustrating how purchasing decisions are increasingly embedded within digitally mediated social interactions. While previous studies have emphasized the importance of electronic word-of-mouth, influencer marketing, and online engagement separately, this study integrates these mechanisms within the broader concept of Evangelism and demonstrates their collective influence on consumer decision-making. The results suggest that consumers do not merely consume marketing content but actively participate in the co-creation and diffusion of brand value through reviews, recommendations, and social sharing.

Third, the study extends understanding of influencer-driven consumption by showing that social validation operates as a key mechanism linking emotional engagement to purchasing behavior. The qualitative findings reveal that consumers rely on influencer content, peer reviews, and community feedback not only to obtain information but also to interpret product value, reduce uncertainty, and construct desired identities. This finding advances existing theories of digital consumer behavior by highlighting the interconnected roles of emotional engagement, identity construction, and social influence within online purchasing processes.

Finally, the study contributes to emerging-market research by providing evidence from Thailand, a rapidly developing social-commerce environment. The findings suggest that consumer behavior in emerging digital markets is increasingly shaped by community-driven interactions and behavioral engagement rather than traditional demographic characteristics. This extends prior research by demonstrating how digitally mediated consumer behavior may be evolving toward behavior-based rather than demographic-based segmentation in highly connected social-commerce ecosystems.

### 5.2. Research Implications

The findings of this study offer several actionable implications for marketers, cosmetics brands, and digital platform managers operating in increasingly competitive social-commerce environments. Given that Evangelism emerged as the strongest predictor of online cosmetics purchase behavior, organizations should prioritize strategies that encourage consumer advocacy and peer-to-peer influence. Rather than relying solely on firm-generated promotional content, marketers should actively stimulate user-generated content through customer reviews, testimonials, referral programs, and social-sharing campaigns. Encouraging consumers to share authentic product experiences can strengthen trust and amplify brand visibility through organic word-of-mouth communication.

The findings also highlight the strategic importance of influencer collaboration. Since consumers frequently rely on influencer recommendations and social validation when evaluating cosmetics products, brands should develop long-term partnerships with influencers whose values and audiences align with their target market. In addition to collaborating with macro-influencers, firms should consider working with micro-influencers, who often possess stronger credibility and closer relationships with their followers. Authenticity and perceived expertise should be prioritized over follower counts alone to maximize consumer trust and engagement.

Furthermore, the significant effect of Experience suggests that marketers should invest in immersive and emotionally engaging digital content. Interactive product demonstrations, short-form videos, storytelling techniques, augmented reality features, and virtual try-on technologies can enhance emotional engagement and strengthen consumer-brand relationships. Live-stream marketing may be particularly effective because it combines entertainment, real-time interaction, product education, and social influence within a single digital environment. By creating opportunities for consumers to interact directly with influencers, brand representatives, and other consumers, live-stream events can increase engagement and reduce purchase uncertainty.

The findings regarding Everyplace emphasize the importance of seamless omnichannel accessibility. Marketers and platform managers should ensure that consumers can easily discover products, compare alternatives, access information, and complete transactions across multiple digital touchpoints. Integrating social media platforms, e-commerce marketplaces, mobile applications, and customer service channels can create a frictionless consumer journey that enhances satisfaction and purchasing continuity.

Finally, although Exchange demonstrated a comparatively weaker effect than the other dimensions, trust-building mechanisms remain essential for sustaining long-term customer relationships. Digital platform managers and cosmetics brands should prioritize transparent product information, verified customer reviews, secure payment systems, responsive customer service, and reliable return policies. These practices help reduce perceived risk and strengthen consumer confidence, particularly in online environments where product evaluation occurs without physical inspection. Collectively, these strategies can enhance consumer engagement, foster advocacy, and improve purchase outcomes in digitally mediated marketplaces.

### 5.3. Limitations and Future Research

Despite its contributions, this study has several limitations. The use of convenience sampling and the focus on consumers in Bangkok may limit the generalizability of the findings to other regions of Thailand and different cultural contexts. In addition, the cross-sectional design restricts the ability to capture changes in consumer behavior over time. Future research could address these limitations by employing probability sampling techniques, including more geographically diverse samples, and adopting longitudinal designs to examine how perceptions of the 4Es and purchasing behavior evolve in response to technological developments and changing market conditions.

Although the model demonstrates strong predictive power, it focuses primarily on the 4Es and selected demographic and behavioral variables. Additional factors, such as psychological traits, brand loyalty, cultural influences, and perceived risk, may further enrich understanding of online consumer behavior and should be incorporated into future research. Furthermore, this study relies on self-reported consumer behavior, which may be affected by social desirability, recall, and common method biases. Because reported attitudes and behaviors may not fully reflect actual purchasing activities, future studies could incorporate behavioral data from digital platforms, transaction records, or observational methods to improve measurement validity.

While the mixed-methods approach provides valuable insights, the qualitative component was limited in scope. Future research could employ larger qualitative samples or alternative approaches, such as netnography and experimental designs, to deepen the understanding of consumer experiences and social influence in digital environments. Comparative cross-cultural studies would also help evaluate the applicability of the 4Es framework across different cultural and market contexts. Such investigations would further strengthen the generalizability and theoretical development of the framework in digital marketing research.

## 6. Conclusions

This study set out to examine how the 4Es framework—Experience, Exchange, Everyplace, and Evangelism—shapes online cosmetics purchase behavior in Thailand, with a particular focus on consumers in Bangkok. In addressing the first objective, the findings demonstrate that all four dimensions exert a significant positive effect on purchase decisions, confirming the relevance of the 4Es as a comprehensive framework for understanding consumer behavior in digital marketing contexts. These results indicate that online cosmetics consumption is influenced not only by transactional considerations but also by experiential, relational, and socially driven factors.

Regarding the second objective, the study confirms the strong predictive power of the proposed model. The integration of the 4Es with behavioral variables—such as platform usage, purchase frequency, and social media engagement—provides a robust explanation of online purchase behavior. The results further reveal that behavioral factors play a more prominent role than several traditional demographic variables, suggesting a shift toward behavior-based segmentation in digitally mature markets.

In addressing the third objective, Evangelism emerges as the most influential determinant of purchase intention, followed by Experience, Everyplace, and Exchange. This finding highlights the dominant role of social influence, brand advocacy, and peer-driven communication in shaping consumer decisions. It underscores the growing importance of consumer participation in value creation, where individuals actively influence others through reviews, recommendations, and social media engagement.

Overall, the findings provide a clear answer to the research question: consumers in the study perceive their online cosmetics shopping experiences as a multidimensional process shaped by emotional engagement (Experience), perceived value and trust (Exchange), convenience and accessibility (Everyplace), and, most importantly, social influence and community interaction (Evangelism). Among these dimensions, Evangelism plays a central role in driving attitudes and purchase behavior, reflecting an increasing reliance on social validation in digital environments.

In conclusion, this study contributes to the growing body of knowledge on digital consumer behavior by validating the 4Es framework in an emerging market context and highlighting the critical importance of socially driven and experiential factors in the online cosmetics industry. The findings offer both theoretical and practical insights, emphasizing the need for marketers to move beyond traditional transactional approaches and adopt more engaging, community-oriented, and experience-driven strategies to effectively influence consumer behavior in today’s digital landscape.

## Figures and Tables

**Table 1:** General data characteristics of the questionnaire respondents.

General Information		Frequency	Percentage
Gender	Male	453	45.3%
	Female	547	54.7%
Age	Below 20 years old	38	3.8%
	20–30 years old	444	44.4%
	31–40 years old	198	19.8%
	41–50 years old	127	12.7%
	Over 50 years old	193	19.3%
Educational level	Lower than bachelor’s degree	162	16.2%
	Bachelor’s degree	672	67.2%
	Higher than bachelor’s degree	166	16.6%
Status	Single	797	79.7%
	Married	164	16.4%
	Divorce	39	3.9%
Income	Less than 15,000 THB	437	43.7%
	15,000–30,000 THB	264	26.4%
	30,001–45,000 THB	186	18.6%
	More than 45,000 THB	113	11.3%
Most frequently used social media platform	Facebook	58	5.8%
	Instagram	102	10.2%
	TikTok	421	42.1%
	X (Twitter)	309	30.9%
	YouTube	110	11.0%
Most frequently used online shopping platform	Facebook Marketplace	58	5.8%
	Lazada	172	17.2%
	Shoppee	456	45.6%
	TikTok Shop	314	31.4%
Online cosmetics purchase frequency	Rarely	61	6.1%
	Sometimes	411	41.1%
	Often	303	30.3%
	Very often	225	22.5%
Recent online cosmetics purchase	Yes	669	66.9%
	No	331	33.1%
Future online cosmetics purchase intention	Very unlikely	6	0.6%
	Unlikely	37	3.7%
	Neutral	141	14.1%
	Likely	465	46.5%
	Very likely	351	35.1%
Total		1000	100%

**Table 2:** Descriptive statistics.

Variables	Mean		Std. Deviation
	Statistic	Std. Error	Statistic
Gender	1.55	0.016	0.498
Age	2.99	0.039	1.223
Educational level	2.00	0.018	0.573
Status	1.24	0.016	0.512
Income	1.98	0.033	1.037
Most frequently used social media platform	3.31	0.031	0.994
Most frequently used online shopping platform	3.02	0.027	0.849
Online cosmetics purchase frequency	3.67	0.029	0.902
Recent online cosmetics purchase	0.67	0.015	0.471
Future online cosmetics purchase intention	3.58	0.025	0.797
Experience	3.52	0.023	0.729
Everyplace	3.49	0.022	0.716
Exchange	3.57	0.022	0.694
Evangelism	3.65	0.021	0.672

**Table 3:** Omnibus test of the model’s performance.

		Chi-Square	df	Sig.
Step 1	Step	562.715	13	0.001
	Block	562.715	13	0.001
	Model	562.715	13	0.001

**Table 4:** The model summary.

Step	−2 Log Likelihood	Cox & Snell R Square	Nagelkerke R Square
1	823.323 ^a^	0.430	0.574

^a^ Estimation converged at iteration 6 (parameter changes < 0.001).

**Table 5:** Classification table for back-testing.

			Predicted		
	Observed		Online Cosmetics Purchasing		Percentage Correct
			No	Yes	
Step 1	Online Cosmetics Purchasing	No	445	65	87.6%
		Yes	92	400	81.3%
	Overall Percentage				84.5%

Note: The cut-off value is 0.500.

**Table 6:** Variables in the model.

		B	S.E.	Wald	df	Sig.	Exp(B)	Action
Step 1 ^a^	H1: GEN	−0.505	0.187	7.295	1	0.007	0.603	Accepted
	H2: AGE	0.097	0.079	1.496	1	0.221	1.102	Rejected
	H3: EDU	0.151	0.220	0.471	1	0.492	1.163	Rejected
	H4: STAT	0.320	0.196	2.660	1	0.103	1.378	Rejected
	H5: INC	−0.946	0.134	49.906	1	0.001	0.388	Accepted
	H6: SMP	0.110	0.151	4.010	1	0.045	1.116	Accepted
	H7: SHP	0.890	0.159	31.248	1	0.001	2.434	Accepted
	H8: FRE	0.675	0.164	16.876	1	0.001	1.965	Accepted
	H9: REC	0.654	0.215	9.279	1	0.002	1.924	Accepted
	H10: EXP	0.804	0.235	11.745	1	0.001	2.234	Accepted
	H11: EVP	0.326	0.359	4.500	1	0.034	1.385	Accepted
	H12: EXC	0.161	0.300	6.300	1	0.012	1.175	Accepted
	H13: EVA	1.740	0.463	14.116	1	0.001	5.696	Accepted
	Constance	−13.288	0.990	180.139	1	0.001	0.000	Accepted

^a^ Variable(s) entered in step 1: Gender (GEN), Age (AGE), Educational level (EDU), Employment status (STAT), Monthly income (INC), Most frequently used social media platform (SMP), Most frequently used online shopping platform (SHP), Online cosmetics purchase frequency (FRE), Recent online cosmetics purchase (REC), Experience (EXP), Everyplace (EVP), Exchange (EXC), Evangelism (EVA). Note: Variables with *p*-values below 0.05 are accepted as significant predictors, while those with *p*-values above 0.05 are rejected as not statistically significant.

**Table 7:** General data characteristics of the interview respondents.

No.	Gender	Age	Occupation	Preferred Online Platforms	Types of Cosmetics Purchased
R1	Male	20	University Student	TikTok	Skincare, Fragrance
R2	Male	29	Office Employee	Lazada	Skincare, Haircare
R3	Male	34	Service Worker	Lazada	Haircare, Fragrance
R4	Male	27	Freelancer	Shopee	Skincare, Body Care
R5	Male	30	Beauty Blogger	TikTok	Body Care, Fragrance
R6	Female	38	Office Employee	Shopee	Makeup, Fragrance
R7	Female	42	Government Officer	Shopee	Skincare, Haircare
R8	Female	20	University Student	Lazada	Makeup, Skincare
R9	Female	31	Content Creator	TikTok	Skincare, Makeup
R10	Female	39	Sales Manager	Lazada	Makeup, Fragrance
R11	Female	29	Freelancer	TikTok	Skincare, Makeup
R12	Female	32	Office Employee	Shopee	Makeup, Fragrance

## Data Availability

The raw data supporting the conclusions of this article will be made available by the authors on request.

## Abbreviations

The following abbreviations are used in this manuscript:

GEN	Gender
AGE	Age
EDU	Educational level
STAT	Employment status
INC	Monthly income
SMP	Most frequently used social media platform
SHP	Most frequently used online shopping platform
FRE	Online cosmetics purchase frequency
REC	Recent online cosmetics purchase
EXP	Experience
EVP	Everyplace
EXC	Exchange
EVA	Evangelism
e-WOM	Electronic word of mouth
IOC	Item objective congruence
CR	Composite reliability
AVE	Average variance extracted
